# The association between interatrial shunt and subtypes of migraine: a Mendelian randomization study

**DOI:** 10.1007/s10072-025-08778-4

**Published:** 2026-01-27

**Authors:** Xiaona Che, Xin Qi, Ziang Kong, Xinqi Li, Lin Na, Yunfei Sun, Wenjing Cui, Jing Chang, Xin Xue

**Affiliations:** 1 Department of Cardiology, The Second Hospital of Jilin University, Changchun City, 130000 China; 2 Clinical Laboratory, The Second Hospital of Jilin University, Changchun City, 130000 China; 3 Department of Cardiology, Xi’an International Medical Center Hospital, Xi’an, 710000 China; 4 Department of Cardiology, Affiliated hospital of jilin medical university, Jilin City, 132000 China

**Keywords:** Mendelian randomization, Causal inference, Migraine, Patent foramen ovale, Migraine without aura, Migraine with aura

## Abstract

**Background:**

Approximately one-quarter of adults have a patent foramen ovale (PFO). Migraine is the second most prevalent neurological disorder. The potential causal relationship between PFO and migraine has been a subject of ongoing debate. This study aims to investigate the genetic association between PFO and migraine.

**Materials and methods:**

This study utilized aggregated data from the Finnish database to explore the genetic causal relationship between PFO as an exposure factor and migraine as an outcome factor. We employed a MR design, with inverse variance weighting (IVW) as the primary analysis method, supplemented by MR-Egger regression. Sensitivity analyses included Cochran Q tests, MR-Egger intercept analysis, leave-one-out analysis, and funnel plots.

**Results:**

After screening, we selected four single nucleotide polymorphisms (SNPs) as instrumental variables (IVs) for the Mendelian randomization analysis. The IVW analysis revealed statistically significant associations between PFO and five subtypes of migraine: overall migraine (OR = 1.0531, *P* = 0.0253), migraine with aura (OR = 1.0809, *P* = 0.0227), triptan use in patients with migraine with aura (OR = 1.0986, *P* = 0.0355), migraine without aura (OR = 1.0906, *P* = 0.0209), and triptan use in patients with migraine without aura (OR = 1.1043, *P* = 0.0220). No evidence of horizontal pleiotropy was detected in the MR-Egger intercept analysis. These findings enhance our understanding of the relationship between PFO and migraine.

**Conclusion:**

This study provides preliminary evidence of a positive causal association between PFO and migraine. The analysis suggests that PFO may be associated with an increased risk of migraine.

## Introduction

Interatrial shunt primarily consist of patent foramen ovale (PFO) and atrial septal defect (ASD). In the general population, the prevalence of PFO ranges from 20% to 34%, while the incidence of ASD is approximately 0.1% [1]. Consequently, the majority of cases involving atrial septal shunts are attributed to PFO. Recent studies have demonstrated that PFO is associated with various conditions, including cryptogenic stroke, paradoxical respiratory-hypoxemia syndrome, decompression sickness in divers, and migraine [2, 3]. Although the underlying pathophysiological mechanisms remain incompletely understood, numerous studies have investigated the relationship between PFO and migraine. The potential benefits of PFO closure surgery for migraine patients remain a subject of considerable debate.

As early as 1998, Del Sette et al. conducted a case-control study that first suggested a potential link between PFO and migraine [4]. In 2000, Wilmshurst et al. reported that PFO closure surgery could alleviate migraine symptoms or reduce their frequency [5]. While several retrospective and case-control studies supported a possible pathophysiological association between PFO and migraine, and found that PFO closure surgery could improve or eliminate migraine symptoms, three randomized controlled trials (MIST, PRIMA, and PREMIUM) failed to conclusively demonstrate significant efficacy of PFO closure surgery in relieving migraine. However, subgroup analyses revealed that in patients with migraine accompanied by aura and cerebrovascular diseases, PFO closure surgery was more effective than pharmacological treatment in improving migraine symptoms [6–8].This suggests that certain subgroups of migraine patients, particularly those with PFO-related migraine, may benefit from PFO closure. A meta-analysis of these three randomized controlled trials further indicated that PFO closure surgery significantly reduced the frequency of migraine attacks and the number of monthly migraine days, although there was no significant difference between groups in completely terminating migraine attacks [9].Currently, the causal relationship between PFO and migraine remains controversial. Given that migraine is a multifactorial disease, controlling its recurrence with a single treatment method remains challenging. Moreover, conclusive evidence establishing a direct causal relationship between PFO and migraine is still lacking.

This study employs the Mendelian Randomization (MR) method to investigate the potential association between PFO and migraine (including its various subtypes), aiming to identify which clinical characteristics of migraine patients may benefit most from PFO closure surgery. Mendelian Randomization (MR) is an instrumental variable analysis technique that uses genetic variants (such as single nucleotide polymorphisms, SNPs) as proxies for exposure factors to determine potential causal relationships between exposure and outcome [10]. Since genetic variations are randomly assigned at conception and are generally unaffected by confounding factors such as the environment, MR can minimize the influence of confounding factors and reverse causality biases, thereby enabling unbiased causal inference. To date, there is limited MR evidence supporting a causal relationship between PFO and migraine. This study considers PFO as the exposure factor and examines migraine, migraine with aura, migraine with aura treated with triptans, migraine without aura, and migraine without aura treated with triptans as outcome variables. This is the first study to explore the causal relationship between PFO and migraine using the MR method, aiming to further elucidate the etiology of migraine and provide new insights for its clinical management.

## Materials and methods

### Study design

**Fig. 1:**
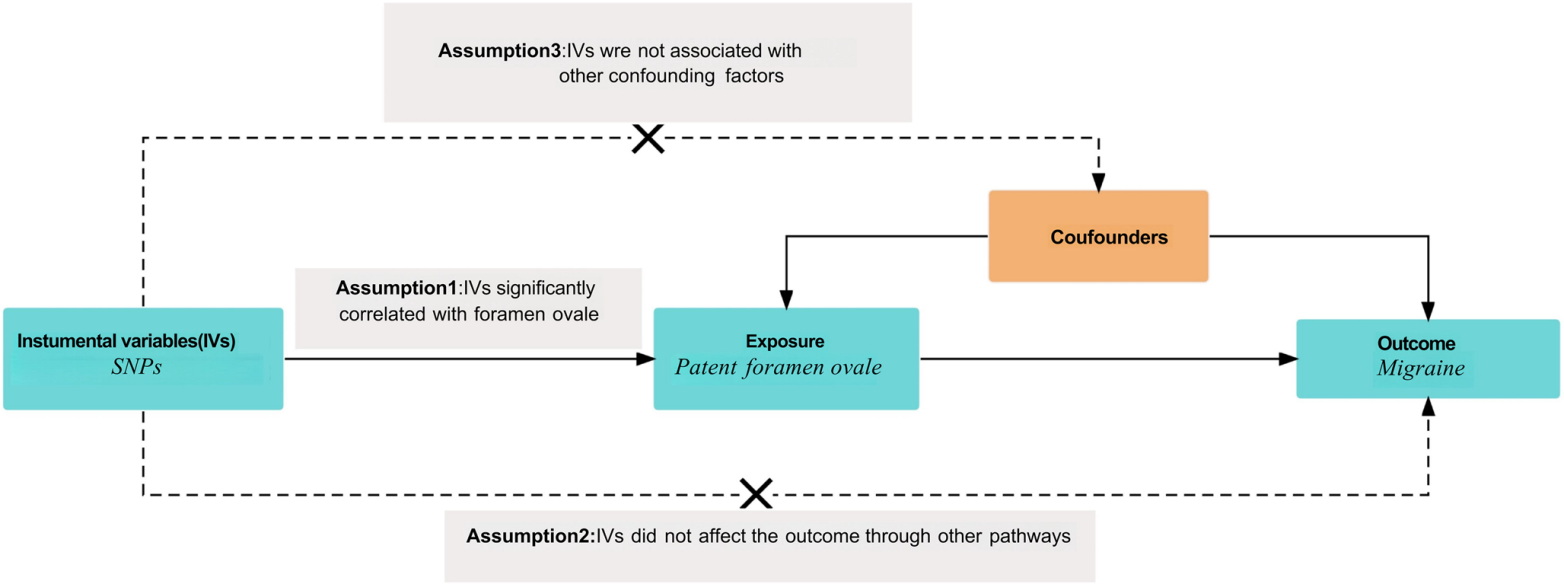
Mendelian randomization research design

Figure 1 illustrates the overall design of our MR study [11]. To ensure the reliability of the MR analysis results, our study strictly adhered to the three core assumptions of the MR method: first, the SNPs used as instrumental variables must be significantly associated with the exposure factor (patent foramen ovale, PFO); second, the instrumental variables should be independent of other potential confounding factors; and third, the instrumental variables should only influence the outcome (migraine) through the exposure factor, without affecting the outcome via alternative pathways. Since our study utilized publicly available published data, no additional ethical review or patient informed consent was required.

### Data sources

The foramen ovale is a channel-like gap that results from the incomplete fusion of the primary and secondary septa during embryonic heart development. This structure typically closes postnatally; however, if it remains open beyond the age of three years, it is termed patent foramen ovale (PFO). Although specific data on PFO were not directly available, we successfully obtained data related to atrial septal defects (ASD) from the Finnish database. According to the International Classification of Diseases, Tenth Revision (ICD-10), ASD is coded as Q21.1, encompassing various conditions such as coronary sinus defect, PFO, secundum-type atrial septal defect (Type II), and sinus venosus-type atrial septal defect. ASD is one of the most common non-cyanotic congenital heart diseases, with an incidence rate of approximately 0.1%. In contrast, PFO is more prevalent, occurring in 20% to 25% of adults [1]. Therefore, the ASD data in the Finnish database likely predominantly represent individuals with PFO. Genetic variation data related to ASD were obtained from the latest FinnGen study (finngen_R12_Q17_ASD), which includes 1,409 cases and 494,430 controls (see Table 1). For more detailed information, please refer to the official FinnGen study website (https://www.finngen.fi/en).

**Table 1:** Sources of GWAS data for instrumental variables

	Dataset	Dataset number	Population	Sample size
Exposure	Atrial Septal Defect	finngen_R12_Q17_ASD	European	1409
Outcome	Migraine	finngen_R12_G6_MIGRAINE	European	26,894
Outcome	Migraine with aura	finngen_R12_G6_MIGRAINE_WITH_AURA	European	11,757
Outcome	Migraine with aura and triptan purchases	finngen_R12_G6_MIGRAINE_WITH_AURA_TRIPTAN	European	6824
Outcome	Migraine without aura	finngen_R12_G6_MIGRAINE_NO_AURA	European	9690
Outcome	Migraine without aura and triptan purchases	finngen_R12_G6_MIGRAINE_NO_AURA_TRIPTAN	European	7324

Migraine is a common neurological disorder characterized by recurrent, typically unilateral, moderate to severe pulsating headaches, often accompanied by nausea, vomiting, photophobia, and phonophobia [12].According to the classification standards of the International Headache Society (IHS), migraine is primarily divided into two main types: migraine without aura and migraine with aura, with approximately 30% of patients experiencing migraine with aura. In this study, genetic variation data related to nine subtypes of migraine were retrieved from the Finnish database. Due to the limited sample size for some subtypes, after screening, data for five subtypes were selected for analysis, including overall migraine, non-aura migraine, non-aura migraine treated with triptans, migraine with aura, and migraine with aura treated with triptans, as detailed in Table 1.Triptan drugs are specifically used to treat migraine attacks and are widely employed in the management of moderate to severe migraine. They help alleviate pain, reduce headache duration, and mitigate associated symptoms. Common triptan medications include sumatriptan, rizatriptan, and zolmitriptan. For more comprehensive information, please refer to the official FinnGen Study website (https://www.finngen.fi/en).

### Instrumental variable selection

To select robust instrumental variables, we first screened SNPs that could predict PFO at the genome-wide significance level (*P* < 5 × 10^−8). We ensured SNP independence by setting an R² threshold of 0.001 and a clustering window size of 1 Mb. After clustering, all SNPs were located in distinct gene regions and distributed independently, without being in linkage disequilibrium. We used Phenoscanner to examine the association between each SNP and potential confounding factors. We calculated the F statistic for each SNP to evaluate its effectiveness as an instrumental variable. The F statistic assesses the strength of an instrumental variable in explaining risk factors and is calculated as F = R²(n-k-1)/k(1-R²). An F statistic greater than 10 indicates that the bias from weak instrumental variables has a minimal effect on the causal relationship. We then mapped the processed SNPs related to atrial septal defects onto the migraine database to extract information about these instrumental variables in the outcome. Finally, we harmonized the data by aligning the statistical parameters of the same loci between the atrial septal defect and migraine datasets, ensuring that the effect sizes of the corresponding effect alleles were consistent across both datasets.

### Statistical analysis

In this study, all bivariate MR analyses were conducted using the TwoSampleMR package in R software (version 4.4.1). The primary analysis methods were the inverse-variance weighted (IVW) approach and MR-Egger regression, employed to evaluate the potential causal relationship between PFO and migraine. Although supplementary methods such as the weighted median were not utilized, IVW remains the most commonly used MR method, assuming all selected instrumental variables (IVs) are valid. This assumption provides high statistical power and relatively accurate results [13]. Additionally, MR-Egger regression was used as a complementary analysis to assess pleiotropy by testing the significance of the intercept term [14]. If all included SNPs satisfy the validity assumptions of instrumental variables, the IVW results can be considered the most reliable [15]. All findings are reported as odds ratios (ORs) with their corresponding 95% confidence intervals, and p-values less than 0.05 are deemed statistically significant.

### Sensitivity analysis

Sensitivity analysis, including heterogeneity and level-dependent multiplicative bias tests, is crucial for assessing the reliability of MR results. For the inverse variance weighted (IVW) method, we applied the Cochran Q test to evaluate heterogeneity. If no significant heterogeneity is detected, a fixed-effects model is used; otherwise, a random-effects model is employed to estimate the MR effect size [16]. Additionally, this study utilized MR-Egger regression analysis to assess potential level-dependent multiplicative bias. The intercept value from the MR-Egger regression reflects the strength of this bias, with a p-value greater than 0.05 indicating no significant level-dependent multiplicative bias [17]. To further verify the robustness and stability of the results, we conducted leave-one-out analyses to examine the impact of removing individual SNPs on the overall findings [18].

## Results

Firstly, by selecting SNPs with a P value less than 5 × 10^−8 and clustering them based on linkage disequilibrium (LD) with an r² threshold of 0.001, we identified four SNPs significantly associated with atrial septal defect. When using the FastTraitR tool to search for the IVs used in the study, it was found that rs117272076 was associated with hair color measurement; however, based on a literature review, this association was considered insignificant and thus was not excluded. All IVs had F statistics greater than 10, indicating high robustness. In the dataset of migraine and its subtypes, no SNPs were excluded, resulting in the final selection of four SNPs as instrumental variables.

**Fig. 2:**
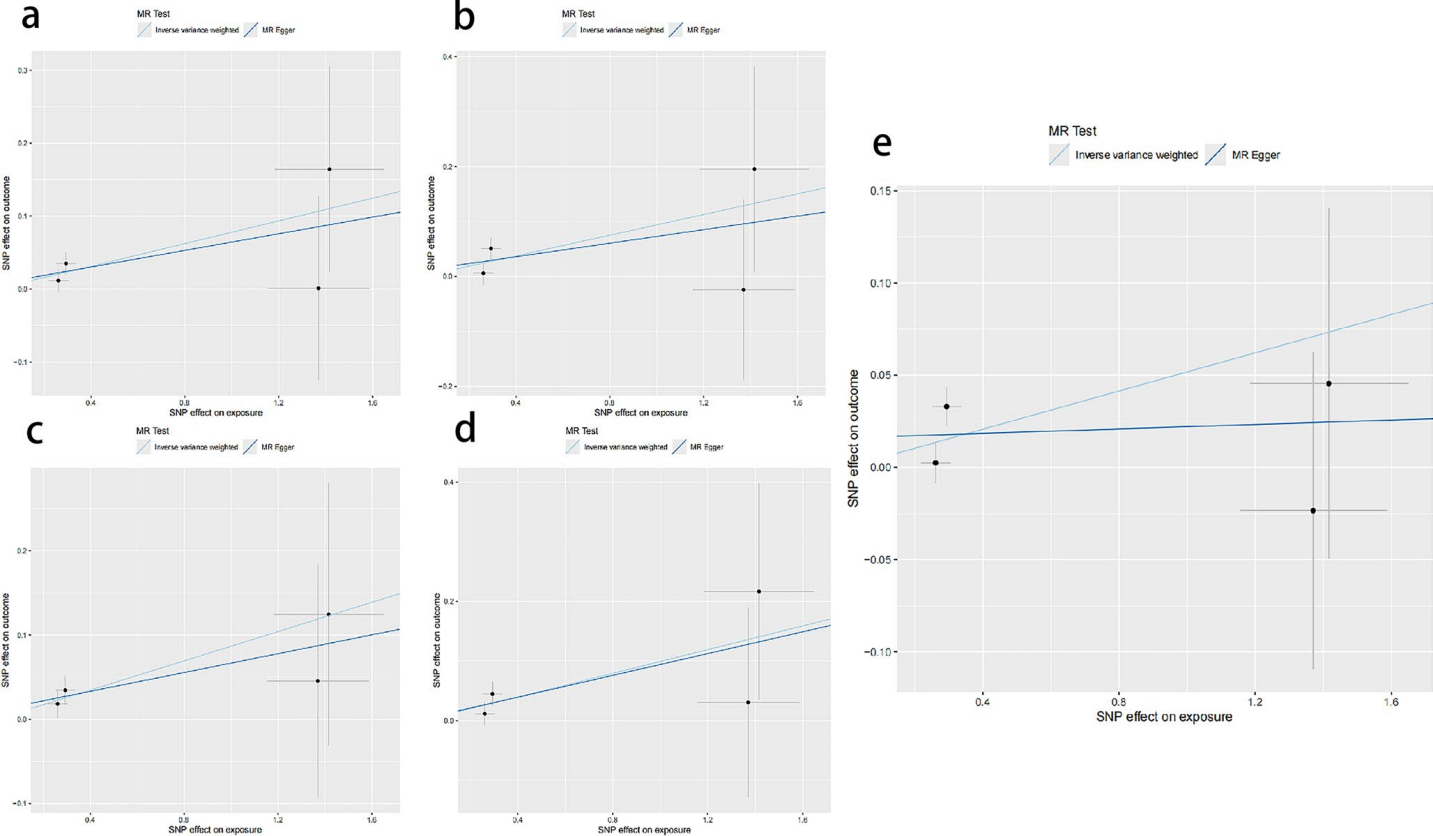
Scatter plots for MR analyses of the causal relationship between patent foramen ovale and various types of migraine. (**A**) Migraine with aura. (**B**) Migraine with aura and triptan purchases. (**C**) Migraine without aura. (**D**) Migraine without aura and triptan purchases. (**E**) Migraine

In this study, the IVW method was systematically employed to analyze the genetic association between PFO and migraine (including its subtypes). The results indicated statistically significant associations between PFO and overall migraine (OR = 1.0531, *P* = 0.0253), migraine with aura (OR = 1.0809, *P* = 0.0227), migraine with aura treated with triptans (OR = 1.0986, *P* = 0.0355), migraine without aura (OR = 1.0906, *P* = 0.0209), and migraine without aura treated with triptans (OR = 1.1043, *P* = 0.0220), as shown in Table 2; Fig. 2. The IVW analysis demonstrated a significant genetic association between PFO and these five types of migraine, suggesting that PFO may be a risk factor for these migraine types and potentially has a positive causal relationship, as illustrated in Fig. 2.

**Table 2:** The IVW analysis of the absence of a relationship between patent foramen ovale and migraine and its subtypes

Classification	β	SE	OR	or-lci95	or-uci95	*P*-val
Migraine	0.051774327	0.023147066	1.053138051	1.006426641	1.102017484	0.025302323
Migraine with aura	0.077827234	0.034148354	1.080935894	1.010956042	1.155759854	0.022661609
Migraine with aura and triptan purchases	0.094042887	0.044735046	1.09860686	1.006382479	1.199282638	0.03553405
Migraine without aura	0.086754559	0.037570557	1.090628962	1.013202663	1.173971976	0.020937461
Migraine without aura and triptan purchases	0.099227941	0.043316826	1.10431799	1.014430084	1.202170798	0.021977983

**Fig. 3:**
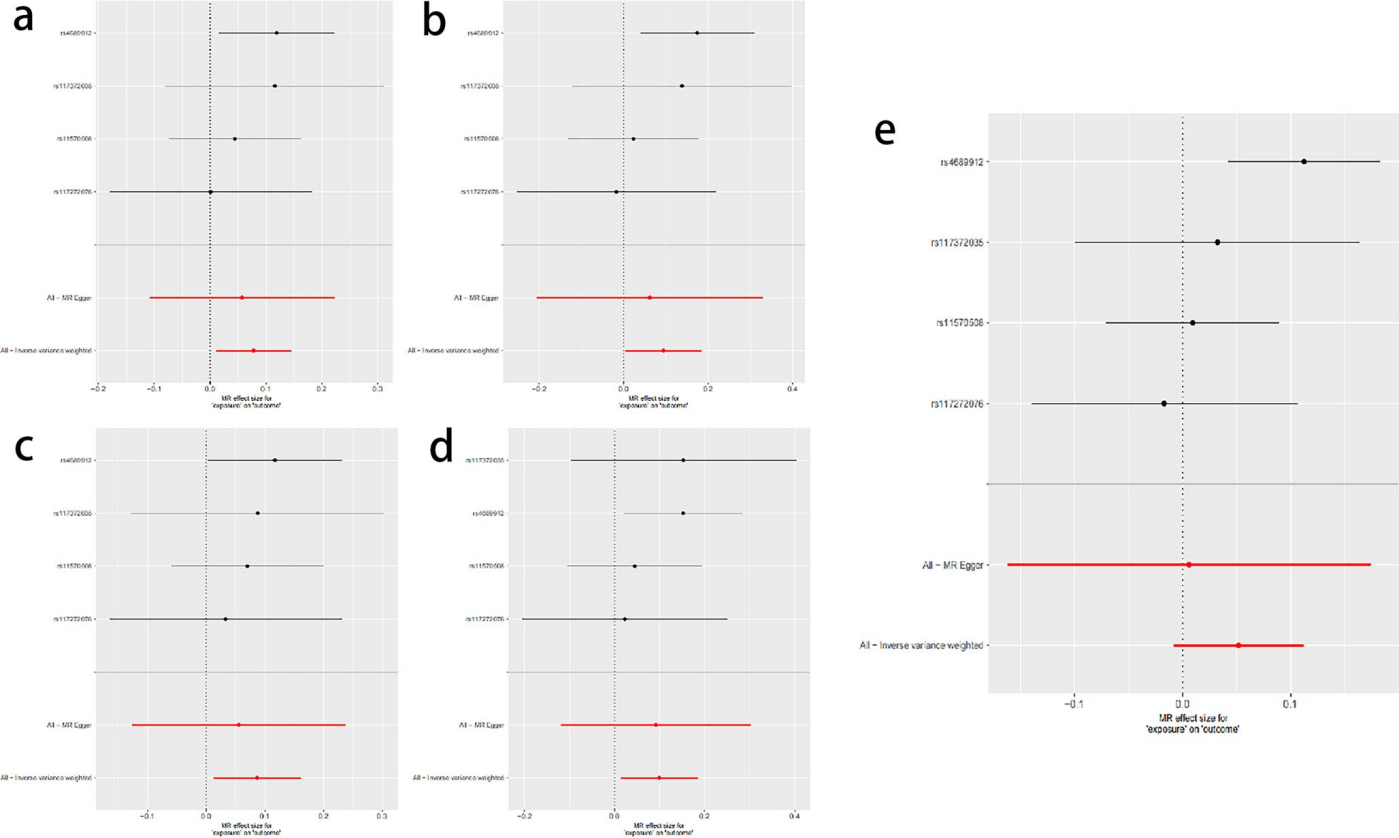
Forest plots for MR analyses of the causal relationship between patent foramen ovale and various types of migraine. (**A**) Migraine with aura. (**B**) Migraine with aura and triptan purchases. (**C**) Migraine without aura. (**D**) Migraine without aura and triptan purchases. (**E**) Migraine

However, the forest plot results indicated that the overall study results for migraine were not statistically significant (see Fig. 3). According to the American Statistical Association’s (ASA) 2016 statement on statistical significance and P-values, P-values alone are insufficient to comprehensively assess the quality of a model or hypothesis. Therefore, although the overall results for migraine did not reach statistical significance, this does not negate the potential causal relationship between migraine and PFO. According to the third edition of the International Classification of Headache Disorders (ICHD-3) released by the International Headache Society (IHS) in 2018, migraine can be classified into six main types: migraine without aura, migraine with aura, chronic migraine (CM), migraine with associated symptoms, probable migraine, and probable related periodic syndromes. In the Finnish database used in this study, migraine was classified as: total migraine, migraine with aura, migraine treated with sumatriptan, migraine without aura, and migraine without aura treated with sumatriptan. We speculate that this overall migraine category encompasses multiple subtypes, some of which may not be related to PFO, potentially affecting statistical power and leading to the non-significance of the overall migraine results. Additionally, the current sample size is limited, and a larger sample size is needed in the future to further verify the stability of the results.

**Fig. 4:**
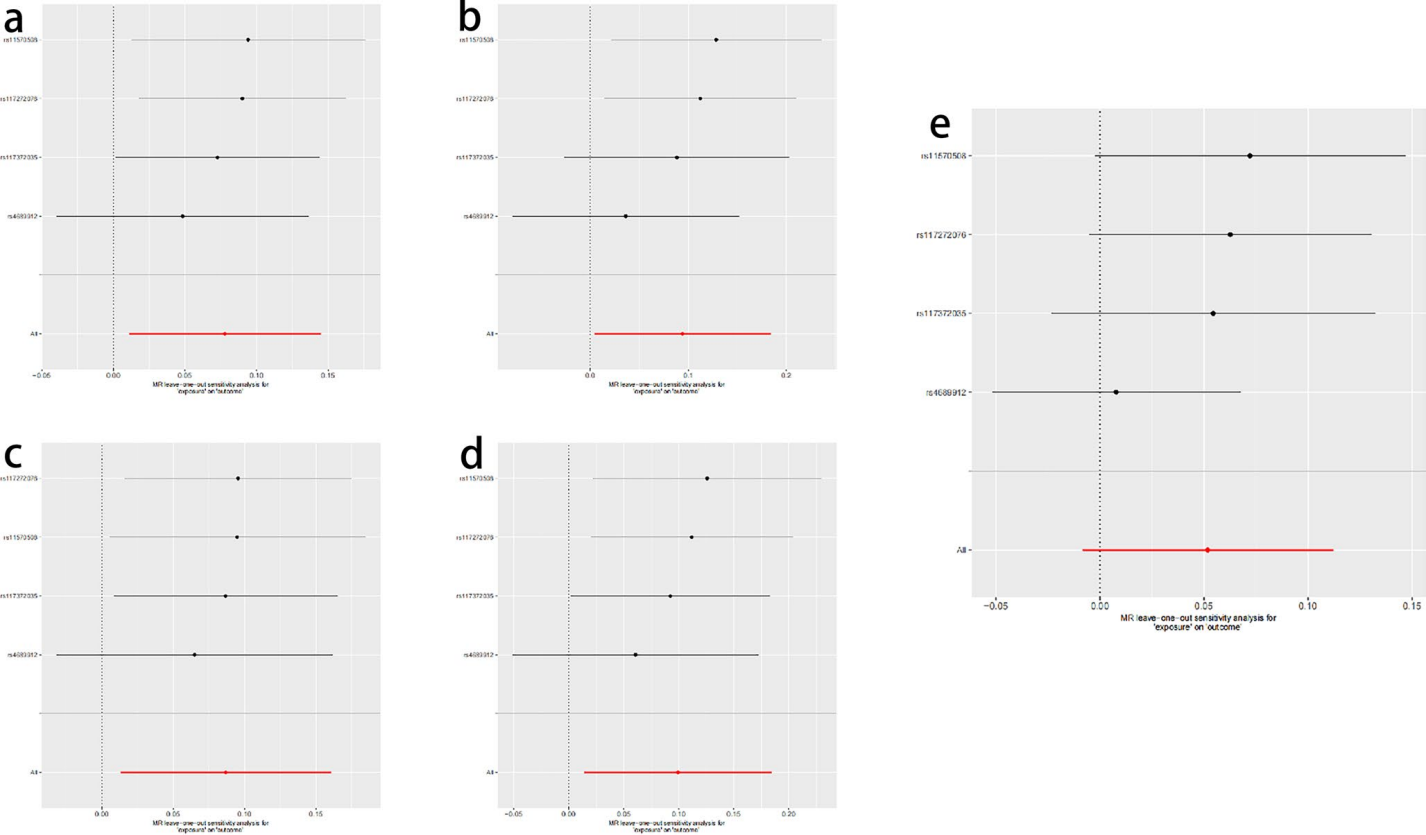
MR leave-one-out sensitivity analyses of the causal relationship between patent foramen ovale and various types of migraine. (**A**) Migraine with aura. (**B**) Migraine with aura and triptan purchases. (**C**) Migraine without aura. (**D**) Migraine without aura and triptan purchases. (**E**) Migraine

**Fig. 5:**
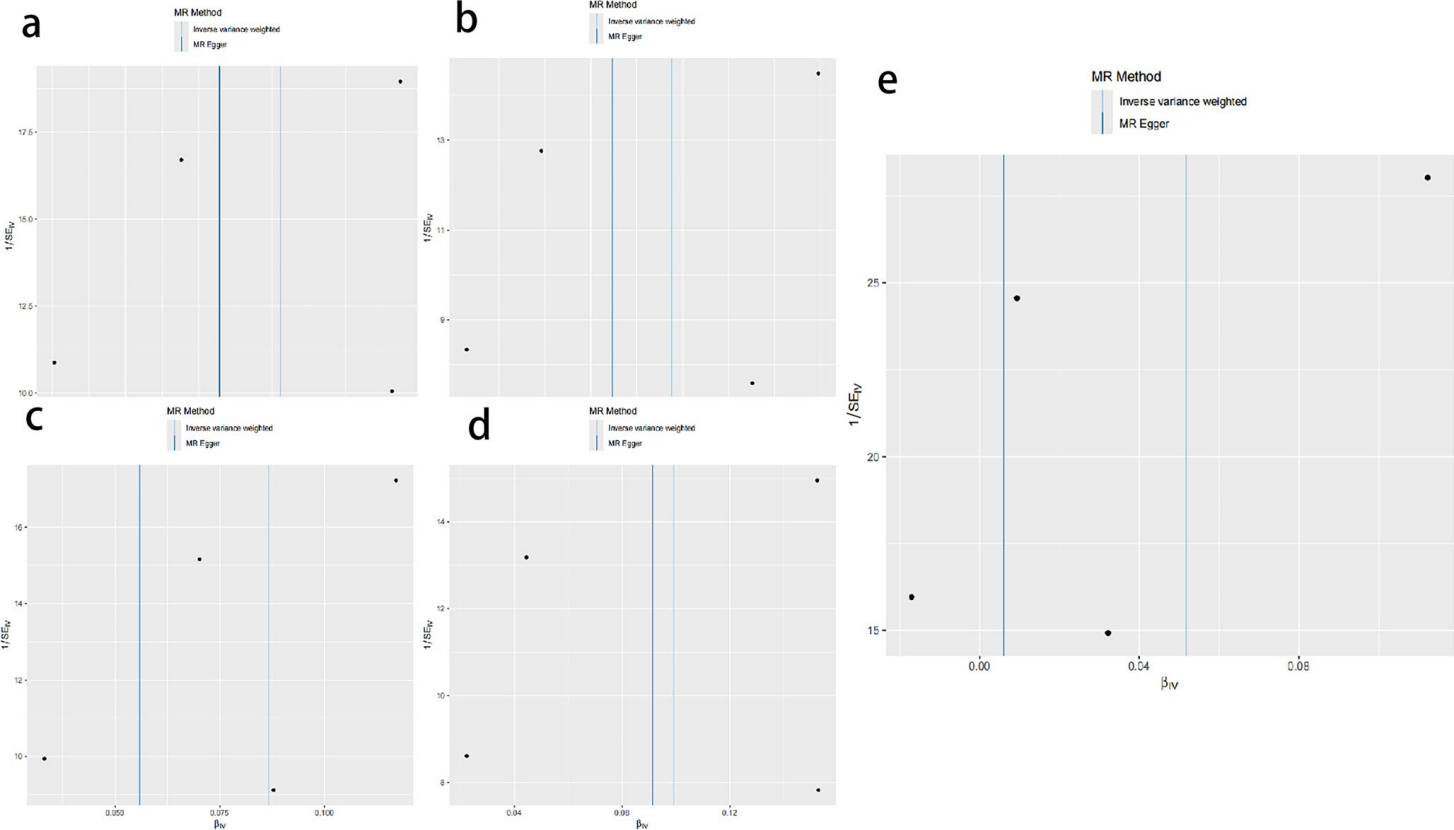
Funnel plots for MR analyses of the causal relationship between patent foramen ovale and various types of migraine. (**A**) Migraine with aura. (**B**) Migraine with aura and triptan purchases. (**C**) Migraine without aura. (**D**) Migraine without aura and triptan purchases. (**E**) Migraine

To verify the robustness and consistency of the results, we conducted multiple sensitivity analyses. The relevant results are detailed in Table 3. The MR-Egger regression results indicated that none of the SNPs for all exposure factors showed horizontal pleiotropy. Heterogeneity tests showed that the P values for all groups were greater than 0.05, suggesting no significant heterogeneity among these SNPs. In the leave-one-out analysis, we verified the stability of the overall estimated results. The results were not driven by a single SNP but reflected the combined effect between atrial septal defect and migraine. Specifically, for the four types of migraine—migraine with aura, migraine treated with triptans, migraine without aura, and migraine without aura treated with triptans—the leave-one-out analysis showed good stability of the results. However, for overall migraine, the analysis results were somewhat unstable. This instability might be due to the fact that the overall migraine category includes multiple subtypes, some of which are not related to the risk factor of patent foramen ovale, and the insufficient sample size also affected the robustness of the results. See Fig. 4. Additionally, the funnel plot results of the Mendelian randomization analysis are shown in Fig. 5.

**Table 3:** Sensitivity analysis of the association between patent foramen ovale and migraine and its subtypes

Exposure	Outcome	Pleiotropy		Heterogeneity	
		Horizontal pleiotropy (Egger intercept)	Horizontal pleiotropy (p-value)	Heterogeneity (Q)	Heterogeneity (p-value)
Atrial Septal Defect	Migraine	0.016	0.618	5.256	0.154
Atrial Septal Defect	Migraine with aura	0.007	0.813	1.768	0.622
Atrial Septal Defect	Migraine with aura and triptan purchases	0.011	0.820	3.138	0.371
Atrial Septal Defect	Migraine without aura	0.011	0.750	0.620	0.892
Atrial Septal Defect	Migraine without aura and triptan purchases	0.003	0.943	1.770	0.622

## Discussion

To the best of our knowledge, this is the first large-scale Mendelian randomization study to assess the causal relationship between PFO and migraine. Our findings indicate that PFO is statistically significantly associated with overall migraine, migraine with aura, migraine with aura treated with triptans, migraine without aura, and migraine without aura treated with triptans. These results suggest that PFO may serve as a risk factor for these types of migraine and potentially has a positive causal relationship.

The relationship between PFO and migraine has long been a subject of controversy. While several studies have indicated an association between PFO and migraine, with higher prevalence of PFO in migraine patients, the results across different studies remain inconsistent. To further investigate this association, three randomized clinical trials evaluated the efficacy of transcatheter PFO closure in patients with migraine.The MIST trial included 432 patients with migraine with aura, of whom 260 (60%) had right-to-left shunt (RLS). The study found that the STARFlex implant did not significantly reduce the frequency of migraine attacks (*p* = 0.51), but it did significantly reduce the total number of migraine days (*p* = 0.027)^6^. The PRIMA trial recruited 705 patients with migraine with aura, of whom 329 (47%) were diagnosed with RLS and PFO. Although the monthly number of migraine days decreased by 1.2 days, this difference was not statistically significant (*p* = 0.17). However, PFO closure significantly increased the response rate (*p* = 0.02), reduced the number of attacks and days, and achieved complete remission in 40% of patients (*p* < 0.01)^7^. The PREMIUM trial included 230 patients with migraine, among whom 8.5% achieved complete remission in the PFO closure group, although it did not meet the primary endpoint (*p* = 0.32). Subgroup analysis showed a significantly higher response rate in patients with frequent migraine with aura (*p* = 0.015), with an increased proportion of complete remission (*p* = 0.04)^8^. A meta-analysis incorporating these three randomized controlled trials demonstrated that PFO closure significantly reduced the average number of migraine attacks (*p* < 0.01) and the monthly number of migraine days (*p* < 0.01). However, there was no significant difference in the proportion of patients who achieved complete cessation of migraine attacks between the two groups (*p* = 0.14)^9^. Another meta-analysis integrating five randomized controlled trials and six observational studies further confirmed that PFO closure surgery significantly reduced the number of migraine attacks per month (*p* < 0.0001) and the number of migraine days per month (*p* = 0.009) [19]. These findings suggest that while there is indeed an association between migraine and PFO, not all migraine patients benefit from PFO closure surgery. This indicates that the etiological mechanism of migraine is complex, and right-to-left shunt is not the sole biological mechanism. However, PFO closure does benefit some patients. Our study further suggests that PFO only modestly increases the risk of migraine, including overall migraine (OR = 1.0531), migraine with aura (OR = 1.0809), migraine with aura treated with triptans (OR = 1.0986), non-aura migraine (OR = 1.0906), and non-aura migraine treated with triptans (OR = 1.1043). These results provide new insights into the potential role of PFO closure surgery in migraine treatment and reveal the multifactorial nature of migraine pathogenesis. They are of great significance for precisely identifying patients who may benefit from PFO closure surgery and support personalized treatment approaches for migraine.

To date, the precise mechanism linking PFO and migraine remains elusive. However, several hypotheses have been proposed, including the microembolus theory, diversion of headache-inducing substances, abnormal cerebral blood flow autoregulation, genetic factors, and platelet activation [20–25]. The prevailing view posits that when PFO induces a right-to-left shunt at the atrial level, microthrombi from the venous system may bypass the pulmonary circulation, enter the left atrium, and subsequently reach the brain via arterial blood flow. This can lead to localized cerebral hypoperfusion and transient hypoxia, ultimately triggering cortical spreading depression (CSD), which is believed to precipitate migraine attacks [20].

Studies have demonstrated that high-resolution optical coherence tomography (OCT) can detect microthrombi within PFO, with in situ thrombi identified in patients with stroke and migraine [26]. An OCT study revealed a significantly higher incidence of in situ thrombi within PFO in the stroke and migraine groups compared to the asymptomatic group [27]. This finding suggests that in situ thrombi may play a crucial role in the pathogenesis of PFO-related stroke and migraine, highlighting their clinical significance and potential as therapeutic targets.Further investigations into the presence and effects of microthrombi have shown that platelet activation is markedly increased in migraine patients, indicating possible primary and secondary coagulation disorders [28–30].Platelet activation not only leads to the formation of platelet-leukocyte aggregates but also promotes the release of inflammatory factors, thereby triggering sterile inflammation in the brain and exacerbating pain signal transmission. Studies have indicated that antithrombotic therapy in PFO patients significantly reduces the risk of stroke recurrence and decreases the frequency and duration of migraine attacks.Our study further verified the statistical association between PFO and migraine, particularly in patients with typical migraine (OR = 1.0986) and those without typical migraine (OR = 1.1043) treated with triptans. Triptan drugs, as 5-HT receptor agonists, are widely used for moderate to severe migraine and effectively alleviate pain, shorten headache duration, and improve related symptoms. Research has shown that in migraine patients treated with triptans, platelet activation levels returned to normal, whereas no such effect was observed in untreated patients [31]. This suggests that triptans may exert a direct inhibitory effect by preventing the transition of platelets from the resting to the activated state.These findings reinforce the potential pathological association between thrombosis and migraine, underscore the role of thrombosis in migraine pathogenesis, and provide robust support for the relationship between PFO and migraine. Our results indicate that for both typical and non-typical migraine patients treated with triptans, the risk ratio is elevated, further substantiating the pathogenic mechanism involving platelets in migraine.

A study published in The Lancet investigated the relationship between migraine and PFO [5]. The research found that for patients with large right-to-left shunts, PFO closure significantly reduced the frequency and severity of aura-related migraines, and also improved symptoms in some patients without aura-related migraines. However, not all patients benefited from the closure procedure, indicating that shunt is not the sole biological mechanism underlying migraine. While PFO closure can reduce the incidence and severity of migraine, this suggests that although PFO is associated with migraine, it is not the core cause for all migraine patients.In subjects with large PFO shunts, subclinical transient cerebral hypoxia/ischemia induced by microbubble injection led to disordered electroencephalogram (EEG) spectra, but headache symptoms occurred only in some patients. Despite similar degrees of right-to-left shunt confirmed by transcranial Doppler (TCD), no comparable EEG activity changes were observed in patients without a history of migraine. This indicates that, in addition to right-to-left shunt, “high sensitivity” of the brain may be a necessary condition for migraine attacks [32]. Our study further demonstrated that PFO only slightly increased the risk of migraine (OR = 1.0531), but the risk was higher in patients with aura-related migraines (OR = 1.0809), particularly in those treated with triptans (OR = 1.0986). For patients without aura-related migraines, the risk also increased slightly (OR = 1.0906), especially in those treated with triptans (OR = 1.1043). These findings suggest that the impact of PFO on different types of migraine patients varies, revealing the multifaceted pathogenesis of migraine.

However, there are several limitations to our study that need to be considered. First, the most fundamental limitation of this study and all trials on the relationship between migraine and PFO to date is that the underlying pathophysiological mechanisms between migraine symptoms and PFO remain unknown. Second, although we were not able to retrieve specific data on PFO directly, we were able to obtain information on atrial septal defects through the Finnish database. According to ICD-10 code Q21.1, this code covers coronary sinus defects, patent foramen ovale defects, second atrial septal defects (type II), and venous sinus defects. According to epidemiological studies, the population incidence of PFO is much higher than other types of atrial septal defect, so we believe that PFO accounts for the vast majority of cases of atrial septal defect recorded in the Finnish database. Finally, the sample size of atrial septal defects in the Finnish database is relatively small. We gradually expanded the data from the 9th edition to the 12th edition. With the increase of sample size, the statistical significance of inverse variance weighting (IVW) method gradually increased. In the future, we hope to obtain larger sample sizes of atrial septal defect genome-wide Association Study (GWAS) data to further validate these findings.Lastly, the same Finnish database was used for exposure and outcome, risking sample overlap. Overlap impact, with a Type I error of 0.05, is minimal, as verified online (https://sb452.shinyapps.io/overlap/).

## Conclusion

This study utilized a two-sample Mendelian randomization (MR) approach to systematically investigate the potential causal relationship between patent foramen ovale (PFO) and various subtypes of migraine. The analysis provided preliminary evidence of a positive causal association between PFO and migraine. Our findings suggest that the impact of PFO on different types of migraine patients may vary, further elucidating the complex pathogenesis of migraine. However, additional experimental validation is necessary to confirm these observations. This study offers novel insights into the potential therapeutic application of PFO closure in migraine management and sheds light on accurately identifying patients who may benefit from PFO closure, thereby advancing the development of personalized migraine treatment strategies.

## Data Availability

We utilized the Finnish database for both exposure and outcome data, with detailed information accessible on the official website of the FinnGen study (https://www.finngen.fi/en).
